# Usefulness of Oral Fluid for Measurement of Methylone and Its Metabolites: Correlation with Plasma Drug Concentrations and the Effect of Oral Fluid pH

**DOI:** 10.3390/metabo13040468

**Published:** 2023-03-24

**Authors:** Giorgia Sprega, Alessandro Di Giorgi, Lourdes Poyatos, Esther Papaseit, Clara Pérez-Mañá, Anastasio Tini, Simona Pichini, Francesco Paolo Busardò, Alfredo Fabrizio Lo Faro, Magí Farré

**Affiliations:** 1 Department of Excellence-Biomedical Sciences and Public Health, Università Politecnica delle Marche, 60126 Ancona, Italy; 2 Department of Clinical Pharmacology, Hospital Universitari Germans Trias i Pujol and Institut de Recerca Germans Trias i Pujol (HUGTiP-IGTP), 08916 Badalona, Spain; 3 Department of Pharmacology, Therapeutics and Toxicology and Department of Psychiatry and Forensic Medicine, Universitat Autònoma de Barcelona (UAB), 08193 Cerdanyola del Vallés, Spain; 4 National Centre on Addiction and Doping, Istituto Superiore di Sanità, 00161 Rome, Italy

**Keywords:** methylone, oral fluid, pharmacokinetics, humans, LC–MS/MS

## Abstract

The aim of this study was to investigate methylone and its metabolites concentration in oral fluid following controlled increasing doses, focusing on the effect of oral fluid pH. Samples were obtained from a clinical trial where twelve healthy volunteers participated after ingestion of 50, 100, 150 and 200 mg of methylone. Concentration of methylone and its metabolites 4-hydroxy-3-methoxy-N-methylcathinone (HMMC) and 3,4-methylenedioxycathinone in oral fluid were measured using liquid chromatography–tandem mass spectrometry (LC–MS/MS). Pharmacokinetic parameters were estimated, and the oral fluid-to-plasma ratio (OF/P) at each time interval was calculated and correlated with the oral fluid pH using data from our previous study in plasma. Methylone was detected at all time intervals after each dose; MDC and HMMC were not detectable after the lowest dose. Oral fluid concentrations of methylone ranged between 88.3–503.8, 85.5–5002.3, 182.8–13,201.8 and 214.6–22,684.6 ng/mL following 50, 100, 150 and 200 mg doses, respectively, peaked between 1.5 and 2.0 h, and were followed by a progressive decrease. Oral fluid pH was demonstrated to be affected by methylone administration. Oral fluid is a valid alternative to plasma for methylone determination for clinical and toxicological studies, allowing for a simple, easy and non-invasive sample collection.

## 1. Introduction

By the information available as of May 2022, a total of 1127 New Psychoactive Substances (NPSs) were reported to the United Nations Office on Drugs and Crime (UNODC) [1]. Due to their heterogeneity, there are several classifying systems, one of these providing a classification in four groups: synthetic stimulants, synthetic cannabinoids, synthetic hallucinogens and synthetic depressants [2]. The largest class of synthetic stimulants is represented by synthetic cathinones, compounds chemically related to cathinone, a psychoactive drug found in the khat plant [3]. Among most incoming abused synthetic cathinones there is 4-methylmethcathinone (mephedrone) and 3,4-methylenedioxy-methcathinone, more commonly known as methylone or MDMC, first identified in 2009 [4]. Its phenethylamine structure is based on that of 3,4-methylenedioxy-methamphetamine (MDMA), with the only difference in the β-ketone group [5]. Both substances inhibit the neuronal reuptake and enhanced the release of dopamine, norepinephrine and serotonin, thus increasing monoamine concentrations in the synaptic cleft [6,7]. Similarly to MDMA, the metabolism of methylone mainly occurs in the liver, where this compound is converted to 3,4-methylenedioxycathinone (MDC) through N-demethylation. However, O-demethylation is another pathway providing 3,4-dihydroxy-N-methylcathinone (HHMC), which is subsequently converted to 4-hydroxy-3-methoxy-N-methylcathinone (HMMC) through O-methylation, the primary metabolite [8,9].

Recently, its acute pharmacological effects after oral controlled administration of 200 mg in comparison to MDMA 100 mg have been published [10]. Methylone pharmacokinetics in humans were firstly published in 2022, in comparison to that of MDMA following controlled administration of different doses [4]. For this purpose, a liquid chromatography–tandem mass spectrometry method for the quantification of methylone and HMMC in plasma was developed. The parent drug and its primary metabolite have also been identified in urine samples from consumers [11], whereas methylone alone was determined in hair from chronic users [12].

Oral fluid (OF) is an alternative biological matrix used to assess current drug consumption in roadside drug testing, used in place of blood for drug monitoring and in pharmacokinetics studies [13,14,15,16]. It allows for a non-invasive, rapid, simple and observed sample collection. Indeed, uncharged basic drugs in blood diffuse across membranes into OF due to the lower pH (6.2–7.4 compared to 7.4 of blood) and ionize, yielding higher OF than blood concentrations [17]. Furthermore, several studies reported the measurement of MDMA and its metabolites in OF as a valuable alternative to plasma determination in clinical and forensic toxicology [18,19].

Concerning methylone, some analytical methods involving gas chromatography–mass spectrometry or liquid chromatography–tandem mass spectrometry have been developed to determine methylone in the OF of cathinone consumers, but no positive samples were found in the analyzed samples [20,21,22,23,24]. Conversely, some other surveys in different parts of the world (United States, Norway, Brazil, Sweden) found OF samples of consumers, reporting a generic use of psychostimulants positive for synthetic cathinones, including methylone [25,26,27,28].

Up to now, no investigation studies on methylone and its metabolites’ time course have been reported for OF after controlled administration.

The aims of this study were:

- To investigate the concentrations of methylone and its metabolites’ MDC and HMMC in OF following controlled administration of different doses to healthy volunteers.
- To assess the eventual correlation between OF and methylone plasma concentrations and to determine the effect of the pH of OF on the methylone OF-to-plasma ratio (OF/P).

## 2. Materials and Methods

### 2.1. Subjects and Study Design

A randomized, cross-over, double-blind, placebo-controlled pilot study was conducted on 12 male volunteers at the Hospital Universitari Germans Trias iPujol, Institut d’Investigació en Ciències de la Salut Germans Trias i Pujol, in Badalona (Spain). All participants had recreational experiences with psychoactive drugs, such as synthetic cathinones, amphetamines, cocaine and MDMA. A general physical examination, a 12-lead electrocardiogram, urinalysis and routine laboratory analyses were performed on each participant. The characteristics of the participants are summarized in Table 1. All subjects declared to be MDMA consumers, with a range of consumption between 5–100 times up to the moment of study, (mean 24). Each volunteer gave written informed consent before the start of the study and was economically remunerated for inconveniences caused by their inclusion in the experiment. The study was conducted in accordance with the Declaration of Helsinki, registered at ClinicalTrials.gov (NCT05488171) and approved by the local Ethical Committee for human research (CEIC-HuGTiP, ref. PI-19-082).

Subjects were divided into four study groups and underwent three administration sessions, with a washout interval of 5–7 days. Specifically, in each session, single oral doses of 50, 100, 150 and 200 mg methylone or a placebo (dextromaltose) were administered to each participant. Lower doses were administered before higher ones. A good tolerability was observed for all doses. Volunteers were requested to abstain from consumption of any drug of abuse during the period of the study. The abstinence was verified by performing urine drug testing before each session. Specifically, the presence of benzodiazepine, MDMA, morphine, tetrahydrocannabinol, methadone, amphetamine, methamphetamine, cocaine, tricyclic antidepressants and barbiturates was tested using the Drug-Screen Multi 10TD Test [Multi-Line] (Nal Von Minden, Moers, Germany). The Pharmacy Service of Hospital Universitari Germans Trias i Pujol (Badalona, Spain) prepared the placebo and methylone as white soft-gelatin capsules (5 capsules each time, combining capsules with active substances and a placebo to reach the methylone dose), which were administered in a fasting state with 200 mL of tap water.

### 2.2. Chemicals

Methylone, MDC and HMMC were supplied from Cerilliant (Round Rock, TX, USA). The internal standard (IS), methylone-d_3_, was purchased from Cayman Chemical (Ann Arbor, MI, USA). Standards were stored at −20 °C until analysis. LC-MS grade water, acetonitrile, methanol, formic acid, ethyl acetate and chloroform were purchased from Carlo Erba (Cornaredo, Italy). In total, 25% purity ammonium hydroxide and 37% purity hydrochloric acid were obtained from Honeywell Fluka™ (Morristown, NJ, USA). Salivette^R^ tubes with cotton swab were purchased from Sarstedt (Nümbrecht, Germany).

### 2.3. Oral Fluid Samples Collection

OF samples were obtained without any stimulation at 0, 0.25, 0.5, 0.75, 1, 1.5, 2, 3, 4, 6, 8, 10 and 24 h after drug administration. Samples were collected using standard Salivette^R^ tubes with a cotton swab and centrifuged. The OF pH was recorded at the time of collection, and the samples were immediately stored at −20 °C until analysis. OF samples from a placebo group that tested negative for methylone were used as drug-free blank samples. Blood samples were obtained at the same time [4].

### 2.4. Sample Preparation

The OF was allowed to thaw at room temperature. A 100 µL sample was fortified with 10 µL of the 100 ng/mL IS solution. After the addition of 2 µL of 2% NH_3_ in H_2_O (pH 9) solution, a liquid–liquid extraction with 2 mL of chloroform/ethyl acetate 9:1 (*v*/*v*) was performed. Samples were roller-mixed for 10 min, centrifuged at 3500 rpm for 5 min and supernatants were separated into clean tubes. A total of 100 µL of 1% HCl in methanol solution (*v*/*v*) was added to avoid evaporative losses, and the samples were dried under a gentle nitrogen stream. Samples were dissolved in 100 µL of chromatographic mobile phase A:B (95:5) and transferred to autosampler vials prior to the injection of 1 µL into the HPLC-MS/MS system.

The concentration of methylone and its metabolites in OF was determined by using a 1290 Infinity II HPLC coupled to a 6470A triple quadrupole mass spectrometer (Agilent Technologies, Palo Alto, CA, USA) equipped with an electrospray ionization source operating in a positive mode. The separation of the compounds was carried out with a Kinetics^®^ 2.6 µm Phenyl-Hexyl column from Phenomenex^®^ (100 mm × 2.1 mm). The readings of 0.1% formic acid in the water and acetonitrile were mobile phases A and B, respectively, and the flow rate was set to 0.4 mL/min. The elution gradient was set as follows: initial conditions were 5% B, held for 1 min, gradually increased to 50% B within 2.0 min, then increased to 95% B within 4.0 min, finally decreased to 5% B and then held for 6 min. The total run time was 6 min. The autosampler temperature was set to 10 °C, and the column oven temperature was 37 °C.

The mass spectrometer operated in the multiple ion monitoring (MRM) acquisition mode, selecting two transitions for each analyte and IS, as validated in our previous study [4].

Validation data are available in the Supplementary Material Table S1.

### 2.5. pH Measurements of Oral Fluid Samples

The pH of the OF samples from the 12 volunteers in the placebo or methylone group was measured at all time intervals using a pH indicator stick (Riedel-de Haën, Hannover, Germany); the range of the pHs was between 6.4–8 (increments of 0.2 pH units). Two observers recorded results, and they were unaware of the treatment conditions.

### 2.6. Pharmacokinetics and Statistical Analysis

The following parameters of the methylone OF concentrations were determined: maximum concentration (C_max_), time to reach maximum concentration (t_max_) and the area under the concentration–time curve from 0 to 10 h (AUC_0–10_) and from 0 to 24 h (AUC_0–24_). Since what is observed in the OF is a disappearance rather than an elimination, we chose to use a disappearance half-life (t_1/2d_) and disappearance constant (K_d_) to describe the considered parameters [29].

AUCs were calculated using the linear trapezoidal rule; the disappearance constant was calculated using the log-linear regression of the three last points with the concentration above the quantification limit. Correlations between the different variables were analyzed using regression analysis. To assess differences in OF pH values between baseline and different times after treatments, we performed an analysis of variance for the repeated measures (for each treatment) and, when significant, a post hoc analysis comparing the baseline with each time point using a Dunnett test. Differences associated with *p*-values lower than 0.05 were considered statistically significant.

## 3. Results

### 3.1. Concentration–Time Profile and Pharmacokinetic of Methylone, MDC and HMMC in Oral Fluid

**Figure 1:**
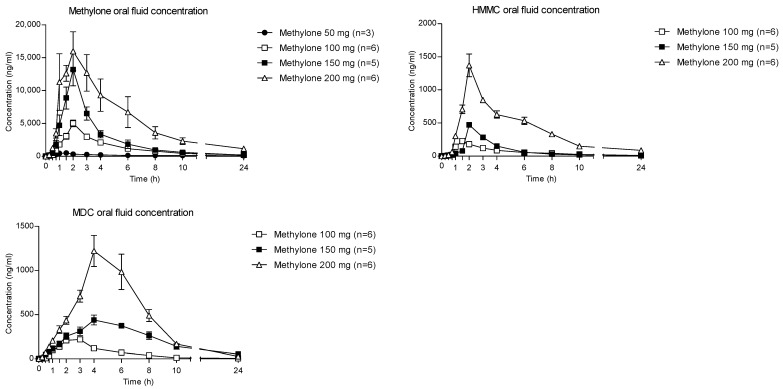
Concentration–time profiles of methylone and its metabolites in OF following controlled administration of different methylone doses.

The time courses of methylone and its metabolites in OF after oral administration of 50, 100, 150 and 200 mg drugs are shown in Figure 1.

The highest concentration of parent compounds in OF was observed at 2 h after each administered dose. Specifically, the means of the methylone C_max_ were reported as 547.8, 5002.3, 13,383.6 and 20,464.9 ng/mL following the 50-, 100-, 150- and 200-mg doses, respectively. After the absorption phase, OF concentrations decreased to mean values of 88 (50 mg dose), 215.7 (100 mg dose), 199.6 (150 mg dose) and 1159.1 ng/mL (200 mg dose) at 24 h after administration.

The pharmacokinetic parameters for methylone in OF are presented in Table 2.

**Figure 2:**
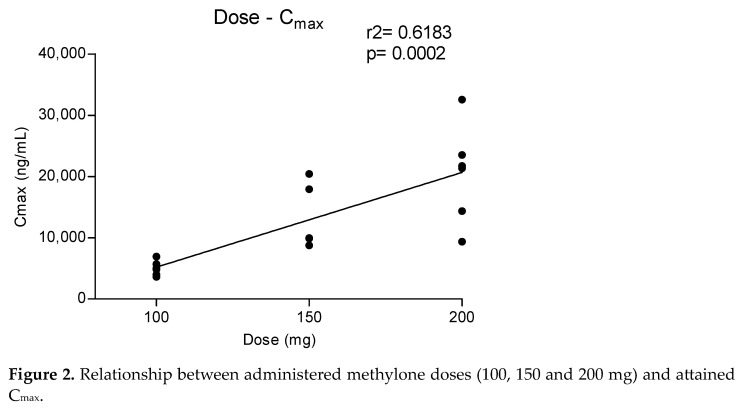
Relationship between administered methylone doses (100, 150 and 200 mg) and attained C_max_.

**Figure 3:**
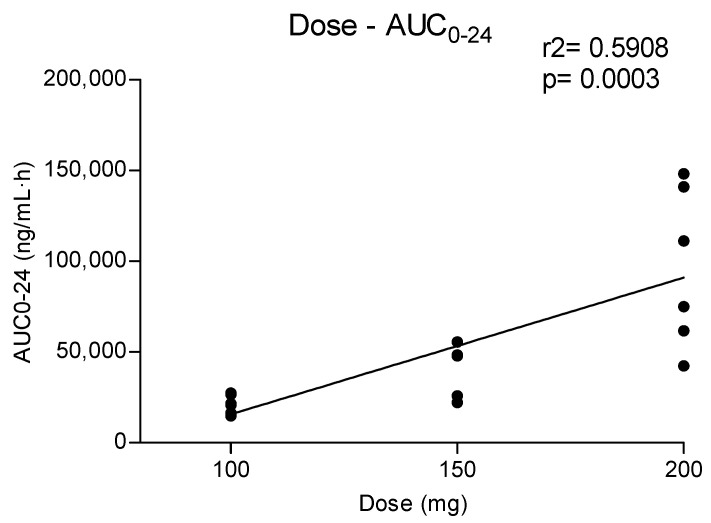
Relationship between administered methylone doses (100, 150 and 200 mg) and obtained AUC_0–24_.

Metabolites MDC and HMMC were not detected following the administration of 50 mg of methylone. Conversely, after the administration of 100-, 150- and 200-mg doses, HMMC reached the C_max_ between 1.5 and 2.0 h and showed AUC_0–10_ values representing 4.6%, 3.2% and 7.2% of the methylone AUC_0–10_ concentrations, respectively. This pattern was similar for AUC_0–24_.The same metabolite reached C_max_ in plasma between 0.9 and 1.5 h [4]. The highest MDC concentration was reached at 3 h after the administration of the 100 and 150 mg doses, while it was reached at 4 h after the 200 mg dose. The MDC AUC_0–10_ was higher than that of HMMC, with values representing 5.4%, 7.9% and 8.7% of the methylone AUC_0–10_ following the 100-, 150- and 200-mg doses, respectively. A similar profile was obtained comparing AUC_0–24_ concentrations. Figure 2 and Figure 3 show the correlation between the methylone dose and the values of C_max_ as well as correlation between the dose and AUC_0–24_, respectively. In both cases, a linear correlation value was observed when considering doses between 100 to 200 mg (r^2^ = 0.6183 and r^2^ = 0.5908, respectively), despite an elevated intersubject variability and the small number of participants. The dose of 50 mg was excluded from the calculation due to the low number of participants administered with this dose (n = 3).

### 3.2. Measurement of pH in Oral Fluid Samples

**Figure 4:**
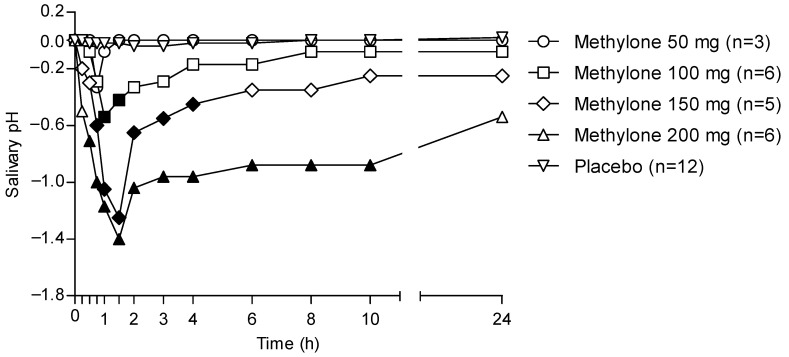
Time profiles of OF pH following controlled administration of different methylone doses. Filled symbols indicate significant differences in that time point with respect to each dose baseline.

Figure 4 shows the 24 h time profile of the OF pH following the administration of different methylone doses and considering placebo samples as baselines.

OF pH decreased in individuals treated with methylone when compared with that from the placebo group. In particular, the pH pattern matched that of methylone pharmacokinetics in OF, showing a minimal decrease for the lowest 50 mg dose, while significant decreases were observed with increasing doses. Minimum pH values were obtained between 1 and 2 h, corresponding to the t_max_ for each administered dose. Finally, the pH returned to pretreatment values only in the case of the 50-mg dose, whereas for the other drug doses, it kept on showing values lower than the pre-treatment ones.

**Figure 5:**
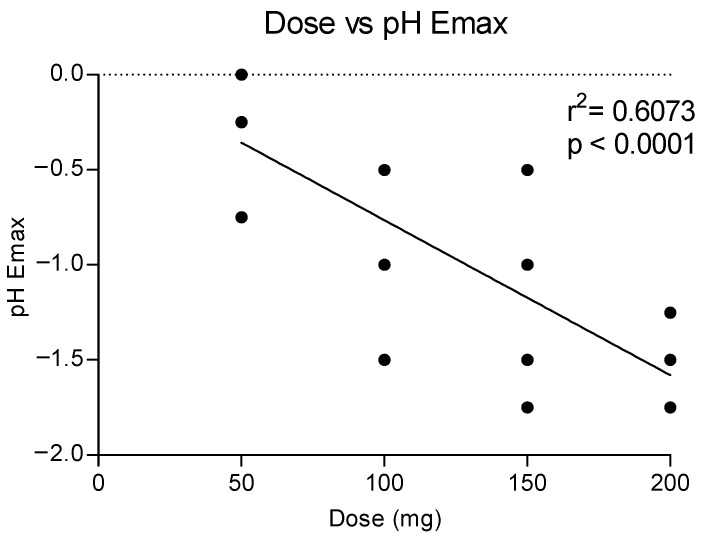
Correlation between methylone doses and maximum effects on pH (Emax).

Figure 5 shows the correlation between the doses of methylone and the maximum effect on the pH, showing a dose response with a linear correlation (r^2^ = 0.6073). Higher doses correlated to a higher reduction in the pH values, as also shown in the time course of pH changes by time and dose (see Figure 4).

### 3.3. OF/plasma Ratio for Methylone

**Figure 6:**
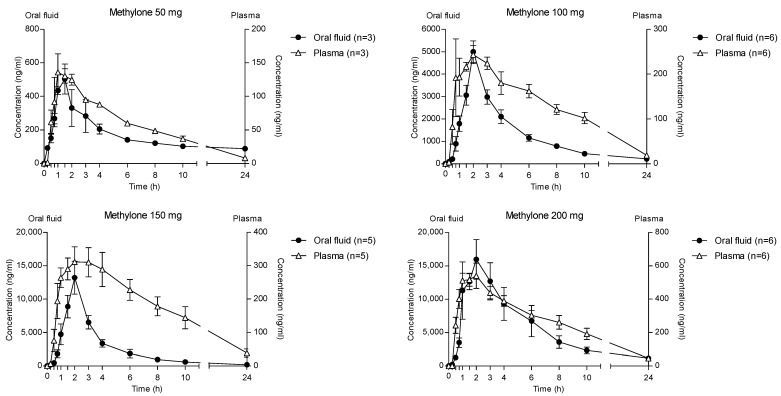
Time profile of different methylone concentrations in OF and plasma.

The time course of different methylone doses in OF and plasma is reported in Figure 6. Methylone reached the highest concentration in both matrices at 2 h after administration of different doses, but the OF values were significantly higher than those in plasma. Specifically, the main ratios between the OF and plasma C_max_ were 3.6, 16.4, 37.7 and 33.9 after the administration of 50, 100, 150 and 200 mg methylone, respectively.

**Figure 7:**
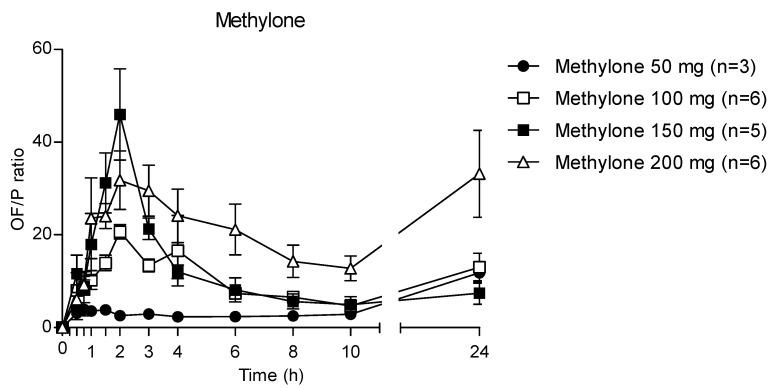
Time profile of methylone OF/P ratio.

Accordingly, after increasing the administered methylone doses, the ratio the 24 h OF/P ratio exhibited mean maximum values of 20.7, 46.0 and 31.8 at 2 h (corresponding to the t_max_) after 100, 150 and 200 mg methylone, respectively, while after 50 mg dose the highest OF/P was 11.8 at 24 h (Figure 7). The *p*-value was <0.0001 for each administration.

## 4. Discussion

For the first time ever, this study showed the pattern of the OF concentration–time profile of methylone and its metabolites HMMC and MDC following increasing parent drug doses.

As expected, methylone appeared in OF at concentrations remarkable higher than those in plasma after each administered dose. As already demonstrated for MDMA [19], this is probably due to the passive diffusion between plasma and OF. Indeed, methylone is a basic drug (pKa 7.96), and the lower OF pH leads uncharged methylone in blood to diffuse across membranes into OF and ionize, accumulating in this matrix (ion trapping phenomenon).

The theoretical OF/P ratio for methylone should be 3.3, as calculated with the Henderson–Hasselbach equation [30]. In our study, the mean OF/P ratio was 3.8, 20.7, 46.0 and 24.1 at t_max_ following 50, 100, 150 and 200 mg of methylone. The difference between the theoretical and experimental OF/P ratio values are attributable to the pH decrease, which matches with the pharmacokinetic time profile of methylone; indeed, methylone OF concentrations increases with the decrease in pH. It should be noted that the OF/P ratio at t_max_ following the 50 mg dose was the only experimental value similar to that obtained theoretically. In support of this thesis, no significant changes in the pH were observed after 50 mg of methylone. Then, the ratio increased exponentially for the 100 and 150 mg doses, showing a nonlinear pattern; finally, it decreases, and after 200 mg it was similar to that obtained after 100 mg of methylone. The mechanism of action may also contribute to an increase in the OF concentrations. Methylone and MDMA act on serotonergic neurotransmission, leading to vasoconstriction and changes in hemodynamics [31], thus the production of OF may be reduced, resulting in a concentration of compounds into this matrix.

In addition, OF contains a considerable amount of inorganic compounds, including water and strong and weak ions (Na+, K+, Mg+, Ca2+, Cl^−^, bicarbonate and phosphate), which may function as buffering agents. We do not know the relevance of the inorganic compound in our study. We tried to standardize all the conditions in order to reduce the relevance of intrinsic/extrinsic factors. In our subjects, only tap water was administered and separated from the OF sampling. Chewing gum, alcoholic beverages or coffee were not allowed during experimental sessions. In addition, subjects presented similar baseline pHs before drug administration. Since methylone shows sympathetic action, the changes in the pH could be the due the norepinephrine action on salivary glands.

The concentrations of methylone in the OF of the present study agree with those observed in a previous observational study, where subjects self-administered different doses of methylone (100 mg, 150 mg, 200 mg and 300 mg; n = 8) and provided OF 1, 2 and 4 h after administration [32].

The exceeding theoretical values were also observed in other studies with methamphetamine after a different route of administration, such as the intravenous route [33]; this allowed us to exclude the hypothesis of buccal contamination for the first 2 h following oral administration. Moreover, similar to our previous study [19], juice and snacks given to the volunteers at 3 h after the administration should have eliminated this contamination also in the following hours.

Overall, the pharmacokinetic profiles showed similar patterns. However, some interindividual variations were noticed, especially for methylone, where CV% increased with increasing doses. As MDMA, methylone was reported to be metabolized by CYP2D6 [34]; its genetic polymorphism may be the cause of high interindividual variations observed in methylone time-course profiles. Furthermore, several xenobiotics are substrates of this enzyme, leading to an inhibition or an induction [35,36].

OF t_1/2d_ was comparable for methylone and HMMC following 100, 150 and 200 mg doses; conversely, MDC showed a different profile in regard to the clearance rate, especially compared to methylone and the HMMC pharmacokinetic profile. In fact, its elimination rate was more rapid following 100 and 200 mg doses, whereas it was slower when the 150 mg methylone dose was administered. This parameter suggests a prolonged effect compared to other synthetic cathinones such as mephedrone (2.12 h) [37]; furthermore, several studies demonstrated that its metabolite MDC can cross the blood–brain barrier in rats, producing a significant increase in brain extracellular dopamine and serotonin concentrations [9]. Therefore, the pharmacological activity of MDC may enhance the psychoactive effects of methylone. However, concentrations were much lower than its parent drug. Conversely, a study demonstrated that HMMC does not cross the blood–brain barrier in rats, thus it may be inactive [8].

## 5. Conclusions

The analytical method demonstrated to be suitable for methylone and metabolite detection in OF, with good precision, accuracy and efficiency. This matrix represents an alternative to plasma for clinical and toxicological purposes, allowing us to detect a recent drug assumption and make a simple, rapid and non-invasive collection. In our study, methylone was detectable at a high concentration (> 80 ng/mL) at 24 h also, but it should be noted that the high administered doses and controlled conditions may have enlarged the detection window. This study confirmed the matching pharmacokinetic pattern of methylone and MDMA, already suggested by the structure similarities. In particular, both compounds exhibited an experimental OF/P ratio much higher than that theoretically calculated through the Henderson–Hasselbach equation. This was evident for higher methylone doses (100, 150 and 200 mg), whereas the two ratios were comparable at the lowest administered dose (50 mg).

## Figures and Tables

**Table 1:** Characteristics of the participants.

	Methylone 50 mg (n = 3)	Methylone 100 mg (n = 6)	Methylone 150 mg (n = 5)	Methylone 200 mg (n = 6)	Placebo (n = 12)
Age (years)	22.3 ± 0.6 (22–23)	22.7 ± 0.8 (22–24)	23.4 ± 0.9 (22–24)	24.0 ± 0.0 (24–24)	23.3 ± 0.9 (22–24)
Weight (kg)	69.7 ± 14.2 (60.4–86.0)	71.0 ± 12.3 (60.4–87.0)	71.9 ± 10.0 (61.9–87.0)	70.0 ± 3.9 (66.7–76.6)	70.2 ± 8.7 (60.4–87.0)
Height (cm)	178.4 ± 3.4 (175.0–181.8)	177.0 ± 2.8 (181.8–174.2)	176.9 ± 4.8 (172.8–185.0)	183.6 ± 9.8 (172.8–193.5)	180.4 ± 7.2 (172.8–193.5)
BMI (kg/m^2^)	21.9 ± 4.5 (18.3–27.0)	22.7 ± 3.9 (18.3–27.9)	23.1 ± 3.7 (19.4–27.9)	21.0 ± 3.3 (18.0–25.7)	21.7 ± 3.5 (18.0–27.9)

**Table 2:** Pharmacokinetic parameters for methylone and its metabolites in OF. Values are presented as mean + standard deviation (SD) and coefficient of variation (CV) %.

	Methylone				HMMC			MDC		
	50 mg (Mean ± SD, CV%) (n = 3)	100 mg (Mean ± SD, CV%) (n = 6)	150 mg (Mean ± SD, CV%) (n = 5)	200 mg (Mean ± SD, CV%) (n = 6)	100 mg (Mean ± SD, CV%)	150 mg (Mean ± SD, CV%)	200 mg (Mean ± SD, CV%)	100 mg (Mean ± SD, CV%)	150 mg (Mean ± SD, CV%)	200 mg (Mean ± SD, CV%)
AUC_0–10_ (ng/mL × h)	2009.3 ± 425.2, 21%	16,724.1 ± 4179.4, 25%	34,454.6 ± 12,481.2, 36%	72,247.7 ± 33,659.2, 46%	778.5 ± 159.1, 20%	1118.0 ± 86.7, 8%	5179.0 ± 583.5, 11%	898.8 ± 95.0, 11%	2735.4 ± 441.3, 16%	6283.7 ± 1491.2, 24%
AUC_0–24_ (ng/mL × h)	3350.0 ± 378.7, 11%	21,353.9 ± 5079.8, 24%	39,993.2 ± 14,995.3, 37%	96,628.3 ± 43,532.8, 45%	1023.1 ± 198.2, 19%	1307.3 ± 91.7, 7%	6821.7 ± 727.1, 11%	1017.5 ± 117.6, 12%	4086.4 ± 1104.4, 27%	7652.2 ± 1611.1, 21%
t_max_ (h)	1.5	2.0	2.0	1.5	1.75	2.0	2.0	2.0	4.0	4.0
C_max_ (ng/mL)	547.8 ± 84.8, 15%	5002.3 ± 1192.7, 24%	13,383.6 ± 5379.8, 40%	20,464.9 ± 7979.5, 39%	236.8 ± 45.3, 19%	473.3 ± 56.4, 11%	1370.6 ± 420.6, 31%	226.2 ± 3.6, 2%	474.4 ± 55.9, 12%	1430.5 ± 383.2, 27%
K_d_ (h^−1^)	0.053 ± 0.022, 42%	0.116 ± 0.02, 17%	0.147 ± 0.01, 7%	0.108 ± 0.02, 18%	0.115 ± 0.01, 9%	0.142 ± 0.01, 7%	0.106 ± 0.02, 19%	0.156 ± 0.02, 13%	0.115 ± 0.05, 43%	0.17 ± 0.03, 18%
t_1/2d_ (h)	14.6 ± 6.2, 43%	6.1 ± 1.0, 16%	4.7 ± 0.4, 8%	6.6 ± 1.5, 22%	6.1 ± 0.5, 8%	4.9 ± 0.4, 9%	6.7 ± 1.0, 15%	4.5 ± 0.6, 13%	7.6 ± 4.6, 60%	4.3 ± 0.9, 22%

Abbreviations: AUC, area under the curve; HMMC, 4-hydroxy-3-methoxymethcathinone; C_max_, maximum concentration; K_d_, disappearance constant; t_1/2 d_, disappearance half-life; t_max_, time to reach maximum concentration, presented as median value; MDC, 3,4-methylenedioxycathinone.

## Data Availability

Data are contained within the article.

## Supplementary Materials

The following supporting information can be downloaded at: https://www.mdpi.com/article/10.3390/metabo13040468/s1, Table S1: Method validation parameters.
