# Supplementary material for: Usefulness of Oral Fluid for Measurement of Methylone and Its Metabolites: Correlation with Plasma Drug Concentrations and the Effect of Oral Fluid pH
Source: Metabolites. 2023 Mar 24;13(4):468. doi: 10.3390/metabo13040468 (PMC10143603; doi:10.3390/metabo13040468)
Supplement: Supplementary file 1 [file metabolites-13-00468-s001.zip › metabolites-2259090-supplementary.pdf]

# Supplementary Material

**Table S1**. Method validation parameters.

| | Linear range (ng/mL) | $r^2$ | p-value | $F_{crit95\%}$ | LOD (ng/mL) | LOQ (ng/mL) | QC (ng/mL) | | | Accuracy (%) | | | Intra-day precision CV (%) | | | Inter-day precision CV (%) | | | Recovery (%) | | | Matrix effect (%) | | |
|---|---|---|---|---|---|---|---|---|---|---|---|---|---|---|---|---|---|---|---|---|---|---|---|---|
| | | | | | | | L | M | H | L | M | H | L | M | H | L | M | H | L | M | H | L | M | H |
| Methylone | 5-100000 | 0.9997 | 0.354 | 0.992 | 0.5 | 5.0 | 15 | 40000 | 80000 | 101.1 | 98.4 | 104.2 | 1.5 | 4.1 | 6.0 | 3.5 | 6.1 | 2.7 | 99.2 | 102.2 | 99.4 | 6.7 | -3.9 | 3.2 |
| HMMC | 0.5-10000 | 0.9998 | 1.435 | 1.634 | 0.3 | 0.5 | 1.5 | 4000 | 8000 | 98.3 | 103.1 | 99.7 | 3.8 | 2.1 | 6.5 | 1.6 | 7.2 | 3.9 | 101.3 | 100.1 | 102.2 | 4.2 | 3.6 | 0.4 |
| MDC | 0,5-10000 | 0.9997 | 0.981 | 0.451 | 0.3 | 0.5 | 1.5 | 4000 | 8000 | 99.2 | 99.5 | 97.3 | 2.9 | 1.7 | 2.4 | 3.5 | 5.3 | 1.3 | 108.1 | 98.1 | 97.4 | 0.3 | -4.3 | -1.5 |

Abbreviations: CV, coefficient of variation; H, high-quality control; HMMC, 4-hydroxy-3-methoxy-N-methylcathinone; L, low-quality control; LOD, limit of detection; LOQ, limit of quantification; M, medium-quality control; MDC, methylendioxycathinone; QC, quality control; $r^2$, correlation coefficient.
